# Modulating asthma–COPD overlap responses with IL-17 inhibition

**DOI:** 10.3389/fimmu.2023.1271342

**Published:** 2023-10-27

**Authors:** Leandro do Nascimento Camargo, Renato Fraga Righetti, Francine Maria de Almeida, Tabata Maruyama dos Santos, Silvia Fukuzaki, Nilo Arthur Bezerra Martins, Miguel Cantadori Barbeiro, Beatriz Mangueira Saraiva-Romanholo, Fernanda Degobbi Tenorio Quirino dos Santos Lopes, Edna Aparecida Leick, Carla Máximo Prado, Iolanda de Fátima Lopes Calvo Tibério

**Affiliations:** ^1^ Faculdade de Medicina FMUSP, Universidade de São Paulo, São Paulo, Brazil; ^2^ Serviço de Reabilitação, Hospital Sírio-Libanês, São Paulo, Brazil; ^3^ Department of Bioscience, Federal University of São Paulo, Santos, São Paulo, Brazil

**Keywords:** asthma, asthma-COPD overlap (ACO), inflammation, anti-IL-17, extracellular matrix remodeling, oxidative stress

## Abstract

**Background:**

IL-17 is a modulator of the inflammatory response and is implicated in lung remodeling in both asthma and chronic obstructive pulmonary disease (COPD). Well as and probably in patients with asthma–COPD overlap (ACO).

**Methods:**

In this study, we evaluated the response of the airways and alveolar septa to anti-IL-17 treatment in an ACO model. Fifty-six male BALB/c mice were sensitized with ovalbumin (OVA group), received porcine pancreatic elastase (PPE group), or both (ACO group). Mice were then treated with either anti-IL-17 monoclonal antibody or saline. We evaluated hyperresponsiveness, bronchoalveolar lavage fluid (BALF) cell counts, and mean alveolar diameter. We quantified inflammatory, response, extracellular matrix remodeling, oxidative stress markers, and signaling pathway markers.

**Results:**

Anti-IL-17 treatment in the ACO anti-IL-17 group reduced the maximum response of respiratory system Rrs, Ers, Raw, Gtis, this when compared to the ACO group (p<0.05). There was a reduction in the total number of inflammatory cells, neutrophils, and macrophages in the BALF in the ACO anti-IL-17 group compared to the ACO group (p<0.05). There was attenuated dendritic cells, CD4+, CD8+, FOXP3, IL-1β, IL-2, IL-6, IL-13, IL-17, IL-33 in ACO anti-IL-17 group in airway and alveolar septum compared to the ACO group (p<0.05). We observed a reduction of MMP-9, MMP-12, TIMP-1, TGF-β, collagen type I in ACO anti-IL-17 group in airway and alveolar septum compared to the ACO group (p < 0.05). We also observed a reduction of iNOS and 8-iso-PGF2α in the airways and in the alveolar septum was reduced in the ACO anti-IL-17group compared to the ACO group (p < 0.05). Regarding the signaling pathways, NF-kB, ROCK-1, and ROCK-2 in the airway and alveolar septum were attenuated in the ACO anti-IL-17 group when compared to the ACO group (p<0.05).

**Conclusions:**

Our results suggest that inhibiting IL-17 modulates cell-associated cytokine production in lung tissue, extracellular matrix remodeling, and oxidative stress in ACO through the modulation of NF-kB and FOXP3.

## Introduction

Asthma and chronic obstructive pulmonary disease (COPD) are distinct obstructive lung diseases. Although both diseases are associated with chronic inflammation and can occur simultaneously in the same patient, their inflammatory processes vary in nature and location. This results in different pathologies, clinical manifestations, and therapeutic responses (1).

In clinical practice and scientific research, asthma and COPD have well-defined diagnostic criteria and pathophysiological mechanisms. Recently, more attention has been paid to a variant with overlapping characteristics of both diseases, e.g., a patient presenting with clinical manifestations of COPD but with a significant response to bronchodilators (2, 3). This has been referred to as asthma–COPD overlap (ACO) (2).

The prevalence of ACO is approximately 1.8% in the general population (4). However, patients are not yet primarily given a diagnosis of ACO, and these patients have been identified in populations with either asthma or COPD. The prevalence of ACO has been estimated to range from 11.1% to 61.0% in patients with asthma and from 4.2% to 66.0% in patients with COPD (5).

Lung remodeling in asthma and in COPD exhibit distinct characteristics. The obstruction in asthma has been thought to primarily affect the large airways, although evidence supporting the involvement of the small airways has emerged (6). In contrast, remodeling of the small airways and lung parenchyma represents the pathology underlying expiratory airflow limitation in COPD (7).

The interleukin 17 (IL-17) family is a group of cytokines that are expressed by T helper 17 (Th17) cells and serve a crucial role in the host defense against extracellular pathogens. (8). IL-17 cytokines are upregulated at sites of inflammation and can interact synergistically with other cytokines, such as TNF-α, to amplify the inflammatory response. These cytokines have been identified as the main mediators of COPD pathophysiology (9). Aside from Th17 cells, other T cell types and innate lymphoid cells also produce IL-17A and can directly modulate lung remodeling.

Other players in lung remodeling are eosinophils, neutrophils, and matrix metalloproteinases (MMPs). Eosinophils and neutrophils can cause epithelial injury and stimulate fibrogenesis through the production of transforming growth factor beta (TGF-β), which regulates collagen production (10). The expression of TGF-β seems to be correlated with the degree of subepithelial fibrosis (11). MMPs can selectively degrade components of the extracellular matrix and play a vital role in the trafficking of inflammatory and structural cells. The dysregulation between MMPs and their inhibitors contributes to tissue damage and several remodeling characteristics observed in both COPD and asthma. (12).

Important intracellular signaling pathways triggered by oxidative stress in asthma and COPD include TGF-β and nuclear transcription factor-κB (NF-κB) signaling. NF-κB expression and signaling activation are elevated in COPD, particularly in airway epithelial cells and macrophages (13). Furthermore, oxidative stress triggers the activation of TGF-β signaling pathways. This, in turn, triggers further oxidative stress and promote small airway fibrosis (14). Additionally, oxidative stress upregulates the expression of MMP9, an elastolytic enzyme critical in emphysema pathogenesis, resulting in enhanced neutrophil elastase activity (15).

In recent years, our research group has investigated the role of Th17 cells in chronic allergic inflammation and pulmonary emphysema. We demonstrated that an increase in IL-17 is associated with airway hyperresponsiveness, increased neutrophil infiltration in the airway and lung parenchyma, and recruitment of inflammatory cells in lung remodeling. (16–18). These processes occur via the activation of the NF-kB and Rho kinase signaling pathways. However, the role of IL-17 in ACO is still uncertain. Hence, it is crucial to elucidate the underlying pathophysiological mechanisms driving inflammation and lung remodeling in ACO. Gaining a deeper understanding of these mechanisms could potentially guide the development of promising therapeutic approaches for ACO, including IL-17 inhibition.

## Materials and methods

Prior to experimentation, this study received ethical approval from the Research Ethics Committee of the University of São Paulo School of Medicine (Protocol No. 1029/2018). We conducted this work in the Laboratory of Experimental Therapy I (LIM 20) of the University of São Paulo Faculty of Medicine.

### Experimental groups

We obtained 56 male BALB/c mice from the animal facility of the University of São Paulo School of Medicine. These animals were used for the induction of asthma, COPD, and ACO models.

All animals in this study received appropriate care following the guidelines outlined in the “Guide for the Care and Use of Laboratory Animals” and “Brazilian guide to the production, maintenance or use of animals in teaching or scientific research activities” (19, 20). At the start of the sensitization protocol, the animals had an average body weight of 20–25g. Throughout the experimental procedure, some animals were lost. These were promptly replaced to consistently maintain a total of eight animals per group.

The experimental groups were the following:

A) **SAL group:** intraperitoneal (i.p.) injection and inhalations of sterile saline solution (SAL) (n = 8).
B) **SAL anti-IL-17 group:** i.p. injection and inhalation of sterile saline solution and treatment with anti-IL-17 monoclonal antibody (n = 8).
C) **OVA Group:** i.p. injection of SAL and inhalation of ovalbumin (OVA) solution (n = 8).
D) **OVA anti-IL-17 group:** i.p. injection of SAL, inhalation of OVA solution, and treatment with anti-IL-17 monoclonal antibody (n = 8).
E) **PPE group:** intratracheal instillation of porcine pancreatic elastase (PPE) (n = 8).
F) **PPE anti-IL-17 group:** intratracheal instillation of porcine pancreatic elastase and treatment with anti-IL-17 monoclonal antibody (N = 8);
G) **ACO group:** i.p. injection of SAL, inhalation of OVA solution, and intratracheal instillation of PPE (n = 8).
H) **ACO anti-IL-17 group:** i.p. injection of SAL, inhalation of OVA solution, intratracheal instillation of PPE, and treatment with anti-IL-17 monoclonal antibody (n = 8).

Our control model was the use of saline (SAL), a model widely employed in various experimental studies. We have previously published both types of controls, both intraperitoneal and intratracheal, and did not observe any differences. Subsequently, we decided to utilize this control model in the current study. It’s worth noting that the OVA model serves as the comparison control for the treatments since our objective is to assess the treatment effects. Therefore, SAL is the ideal control for OVA. SAL + SAL is preferred because the inhalation challenge following intraperitoneal saline administration may represent a form of sensitization, albeit partial. Consequently, SAL + OVA can function as an OVA model (18, 21, 22).

### Experimental asthma model

**Figure 1:**
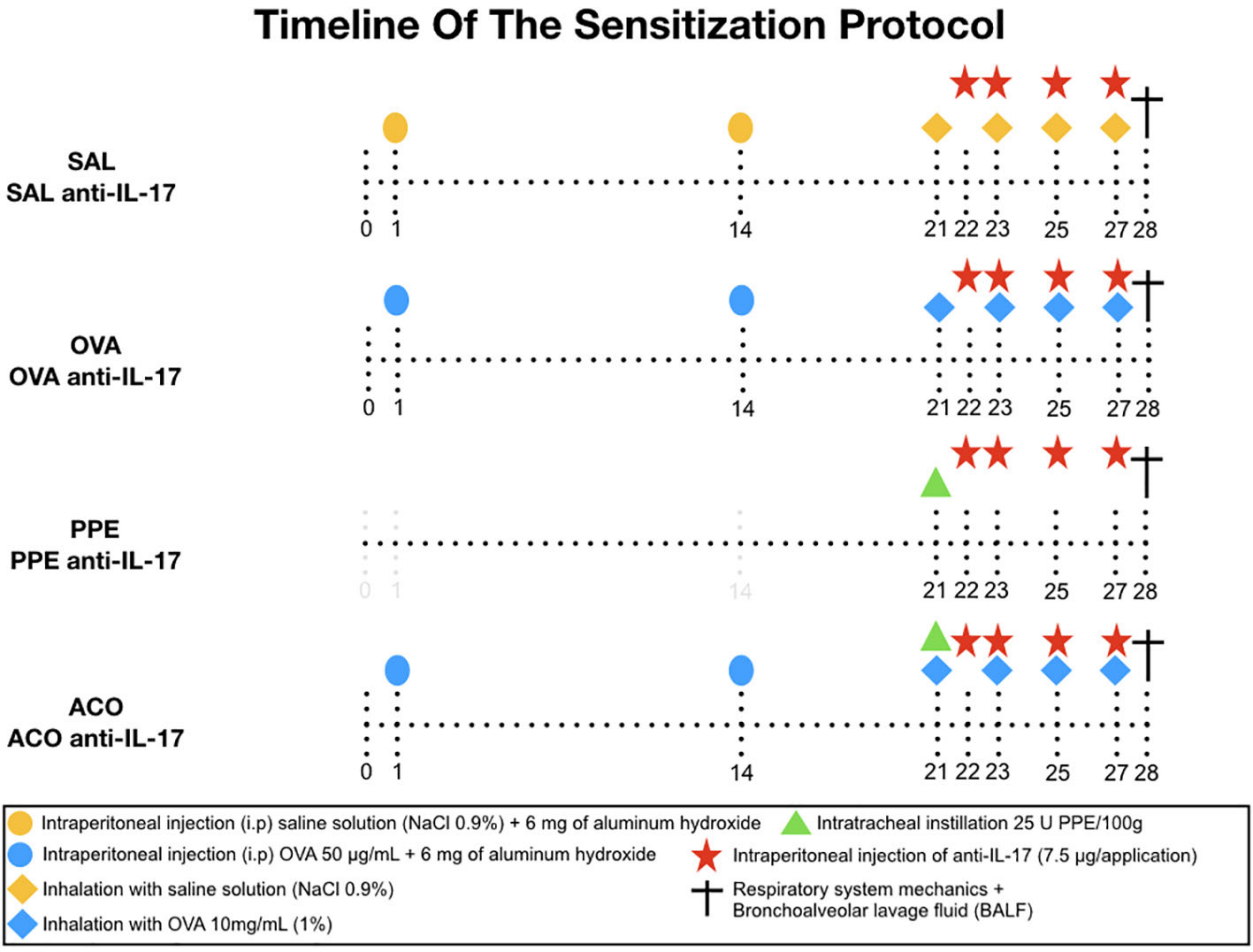
The sensitization protocol was conducted for 28 days. On days 1 and 14, BALB/c mice received intraperitoneal (i.p.) injection of OVA solution. On days 21, 23, 25, and 27, the animals were exposed to 1% OVA aerosol for 30 minutes. The control group received intraperitoneal saline solution and 0.9% saline solution aerosol. PPE instillation was performed intratracheally on day 21. Treatment groups were given neutralizing anti-IL-17 antibody intraperitoneally on days 22, 23, 25, and 27. On day 28, respiratory system mechanics measurements and bronchoalveolar lavage collection were performed.

Sensitization and induction of lung inflammation with ovalbumin were performed over 28 days. Mice received a solution of 50 mg ovalbumin (Sigma-Aldrich) and 6 mg aluminum hydroxide (Alumen, Pepsamar, Sanofi-Synthelabo S.A., Rio de Janeiro, Brazil) i.p. on days 1 and 14. On days 21, 23, 25, and 27, the animals were placed in an acrylic exposure box attached to an ultrasonic nebulizer (US-1000, ICEL, São Paulo, Brazil) and subjected to inhalation of aerosolized 10 mg/ml (1%) OVA in 0.9% saline solution. The duration of exposure to the aerosol was 30 minutes. The control group received 0.9% saline solution and 6 mg aluminum hydroxide (Alumen) i.p. and were exposed to 0.9% saline aerosol for 30 minutes on the days of inhalation challenges. The sensitization procedure is illustrated in **Figure 1** (6).

### Experimental COPD model

The animals were subjected to isoflurane anesthesia (Isofurine^®^ 1 mL/mL, Cristália LTDA, Itapira, SP, Brazil), followed by the preparation of the neck region, which involved shaving and disinfection using Povidone. A mid-neck incision, approximately 0.5 cm in length, was made to expose the trachea. Intratracheal administration of Porcine Pancreatic Elastase (PPE) (EMD Chemicals, San Diego, CA) was performed on the 21st day of the experimental protocol, placed between the cartilaginous tracheal rings. PPE was administered at a dose and concentration of 25 units of PPE per 100 grams of body weight, dissolved in 40 μL of saline solution. Subsequently, the cervical incision was sutured with 5.0 silk and disinfected once again using Povidone. At the end of the procedure, tramadol 60 mg/kg was administered to control pain (23).

### ACO experimental model

Mice in the ACO group were subjected to the procedures described in the experimental asthma and COPD models, following the dose of reagents and schedule indicated above (23, 24).

### Anti-IL-17 treatment

Neutralizing anti-IL-17A (clone50104) antibody (R and D Systems, Abingdon, UK) was administered i.p. on days 22, 23, 25, and 27 of the experimental asthma protocol. The anti-IL-17 was administered 1 hour before the inhalation of aerosolized OVA or SAL, except on day 22 when there was no inhalation.The dose used was 7.5 µg/application. (Camargo 2020). In the pilot study, we had a control-treatment group (SAL anti-IL-17) that received treatment with anti-IL-17 on days 22, 23, 25, and 27. There was no difference between the SAL and SAL anti-IL-17 groups. Therefore, we used the SAL group as a control.

### Determination of exhaled nitric oxide and evaluation of lung hyperresponsiveness to methacholine

Twenty-four hours after induction of experimental disease models, animals were anesthetized with thiopental (50 mg/kg i.p.) and underwent tracheostomy. A plastic cannula was inserted and wired through the tracheostomy hole. Animals were connected to a mechanical ventilation device designed for small animals (FlexiVent, Scireq, Montreal, Canada). Ventilation settings were tidal volume of 10 mL/kg, respiratory rate of 120 cycles/min, and a sinusoidal inspiratory flow curve. To abolish ventilatory effort, animals received pancuronium (0.2 mg/kg i.p.). Gas was collected in the expiratory portion of the ventilator using a collection bag impermeable to nitric oxide (Mylar Bag, Sievers, Instruments Inc., Boulder, CO, USA). After collecting the exhaled NO balloon, the animals were evaluated for their hyperresponsiveness to methacholine (21).

The generated pressure values were measured. Airway impedance (Pressure/Flow) was calculated as a function of the different frequencies produced. Using a 75% overlapping window on the 16-second signal, three 8-second blocks were used to calculate the oscillatory mechanics parameters, using the equation: Z(f) = R_aw_ + i(2 πf)L_aw_ + [G_tis_ = iH_tis_]/(2 πf). These values were used to calculate the respiratory system basal and maximum resistance (R_rs_) and elastance (E_rs_) after methacholine aerosol (3, 30, and 300 mg/ml for 1 min) administration. The resistance and elastance of the respiratory system were calculated using the equation of respiratory system movement. We described the lung model using the following parameters: resistance of larger airways (R_aw_), resistance of smaller airways or tissues (G_tis_), and elastance of lung tissues (H_tis_) (21). After respiratory mechanics evaluation, animals were euthanized by abdominal aorta exsanguination (17).

### Analysis of bronchoalveolar lavage fluid

Following exsanguination of the abdominal aorta, bronchoalveolar lavage (BAL) was performed. Using a syringe, 0.5 mL saline solution (0.9% NaCl) was instilled three times through the tracheostomy cannula, resulting in a total recovered volume of approximately 1.2 mL. The BAL fluid (BALF) was centrifuged at 790 × g for 10 minutes at 5°C.

The cell pellets were resuspended in 300 μL of sterile physiological saline, and 100 μL of each sample was cytocentrifuged in a Cytospin for 6 minutes at 450 rpm. Total cell counts were performed using a Neubauer chamber. For differential cell counts, cytospin slides were prepared and stained with Diff-Quick Reagent (Biochemical Sciences Inc., Swedesboro, NJ). The differential cell count was performed based on 300 cells per slide, following hematological criteria for the differentiation of eosinophils, macrophages, lymphocytes, and neutrophils, using an optical microscope with a 1000x immersion objective, as described by dos Santos et al. (25).

### Histology and immunohistochemistry

After fixation, samples underwent standard histological procedures involving paraffin embedding to obtain sections of 4-µm thickness, followed by staining with hematoxylin and eosin (HE).

**Figure 2:**
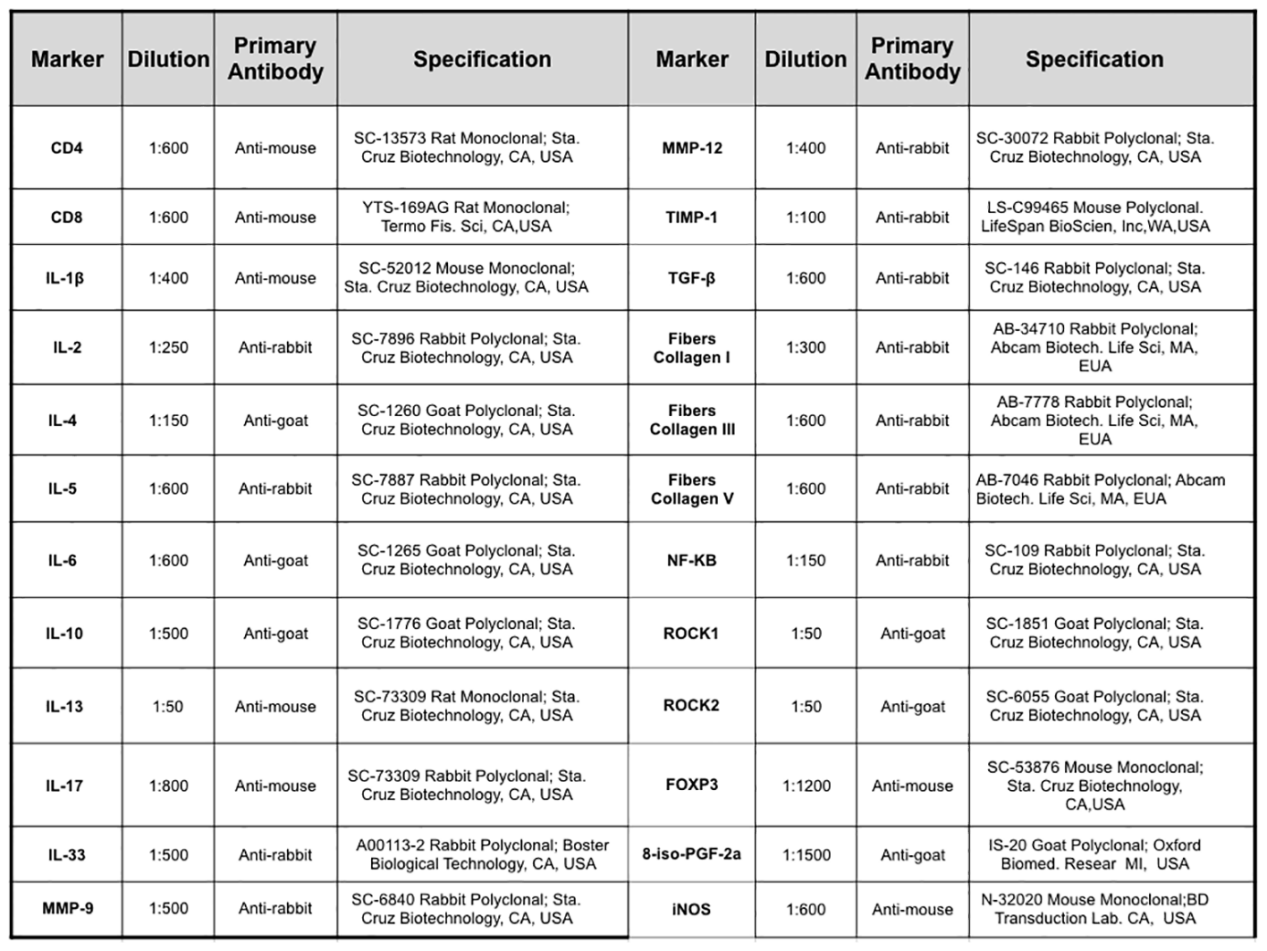
Description of the primary antibodies used in the immunohistochemical staining of the lung samples.

Picro–Sirius staining was performed to visualize collagen fibers. The sections were deparaffinized and immersed in water. Samples were subsequently stained with Picro–Sirius solution at room temperature for 1 hour, followed by a 5-minute wash in running water. Next, the sections were stained with Harris hematoxylin for 6 minutes and washed in running water for 10 minutes (24). To prepare the samples for immunohistochemistry, the sections underwent a series of steps, including deparaffinization, hydration, digestion, and antigen retrieval. For the detection of 8-iso-PGF2α expression, 100 μL of a solution containing 0.25% trypsin (porcine pancreatic trypsin, Sigma Aldrich, Missouri, USA) was applied to the lung sections and maintained for 20 minutes at 37°C. For other markers, the sections were subjected to steam exposure in a pressure cooker (Dako Cytomation, California, Inc.) for 1 minute at 125°C, with the slides immersed in a citrate solution at pH 6 (Target Retrieval Solution, DAKO, California, USA). Following this step, the slides were washed with running and distilled water (3 minutes each). Subsequently, peroxidase blocking was performed using 3% hydrogen peroxide for 5 minutes, followed by three washes with PBS. For the 8-iso-PGF2α antibody, blocking was performed using 10V 3% hydrogen peroxide and methanol (2x for 10 minutes each). Diluted antibodies (**Figure 2**) were pipetted onto the sections, and the slides were incubated in a humid chamber overnight (18-20 h). On the subsequent day, the slides were rinsed with PBS and subjected to incubation with a secondary antibody from the ABCKit by Vectastain system (Vector Elite-PK-6105 anti-goat, PK-6101 anti-rabbit, and PK-6102 anti-mouse). To visualize immunopositive cells, slides were washed with PBS and proteins were visualized using 3,3′-diaminobenzidine (DAB) chromogen (Sigma Chemical Co., St. Louis, MO, USA). The slides were counterstained with Harris hematoxylin (Merck, Darmstadt, Germany). Finally, the slides were mounted using Entellan microscopy resin (Merck) and subjected to morphometric analysis as described below (17, 26).

### Morphometric analysis

#### Linear Mean Intercept

. LM measurements were conducted on slides stained with HE, using a microscope (E200MV, Nikon Corporation, Tokyo, Japan) at 400x magnification. A crosshair with a known area (50 lines and 100 points) was used to determine the number of intersections with the alveolar walls. The initial area of the alveolar walls, excluding the airways, was determined. For each animal, 20 randomly selected lung parenchyma fields were analyzed. The mean alveolar diameter was calculated using the length of the distal parenchyma as reference. The number of intersections between the crosshair lines and the alveolar walls was counted. All LM values were expressed in micrometers, following the methodology described previously (27). LM is an indicator of the average diameter of the distal air spaces and serves as surrogate measure for the degree of alveolar distention.

#### Positive cell counting in the airway and alveolar septum

Morphometric analysis was performed with an optical microscope (E200MV, Nikon Corporation, Tokyo, Japan). A crosshair of lines and points (50 lines and 100 points) was attached to the eyepiece, totaling an area of 10^4^ µm^2^. Counting was performed using the point counting technique and straight lines (28). For the airway analysis, the reticle was positioned along the bronchoalveolar axis, starting from the base of the epithelium. Three airways were randomly selected in each animal, and three fields within each airway were assessed. The number of points coinciding with positive cells on the airway wall was divided by the total number of points. Regarding the alveolar septum, the reticle was attached to this region, and ten random fields were chosen for evaluation. For the septal analysis, the number of points that coincided with positive cells on the alveolar walls within the reticle was divided by the total number of points coinciding with the alveolar walls. All measurements were performed at 1,000× magnification (28).

#### Evaluation of images to measure optical density

Optical density was measured to assess the volumetric fractions of total collagen fibers, collagen types I, III, and V, as well as the isoprostane PGF2α. Microscopic images were captured using a Leica DM2500 digital camera (Leica Microsystems, Wetzlar, Germany). The Optimas v.4.10 software was employed for image processing and analysis. Three airways and ten fields for the alveolar septum were analyzed per slide, with one slide per animal. Image analysis was performed using Image-Proplus 4.5 software (NIH, Maryland, USA). This software facilitated the determination of a color tone threshold and allowed for the quantification of positive areas within the predefined total area. The volume fraction of positive areas was calculated by dividing it by the total area, and the values were expressed as percentages (6).

#### Evaluation of cytokines in lung tissue

IL-6, IL-17, and TGF- β levels in the bronchoalveolar lavage fluid were quantified using an ELISA kit (Duo Set, R&D Systems, Minneapolis, MN, USA). The immunoassay was performed following manufacturer’s instructions, as previously described by Bittencourt-Mernak et al. (29).

### Data analysis

All analyses were performed using SigmaPlot 11.0 software (Systat Software, SPSS Inc., USA). Results are presented as means ± standard error (SE). Differences among treatment groups were determined using One-Way Analysis of Variance with *post hoc* Holm–Sidak test for multiple comparisons if there were significant main effects. A p-value < 0.05 was considered statistically significant.

## Results

### Airway hyperresponsiveness

**Figure 3:**
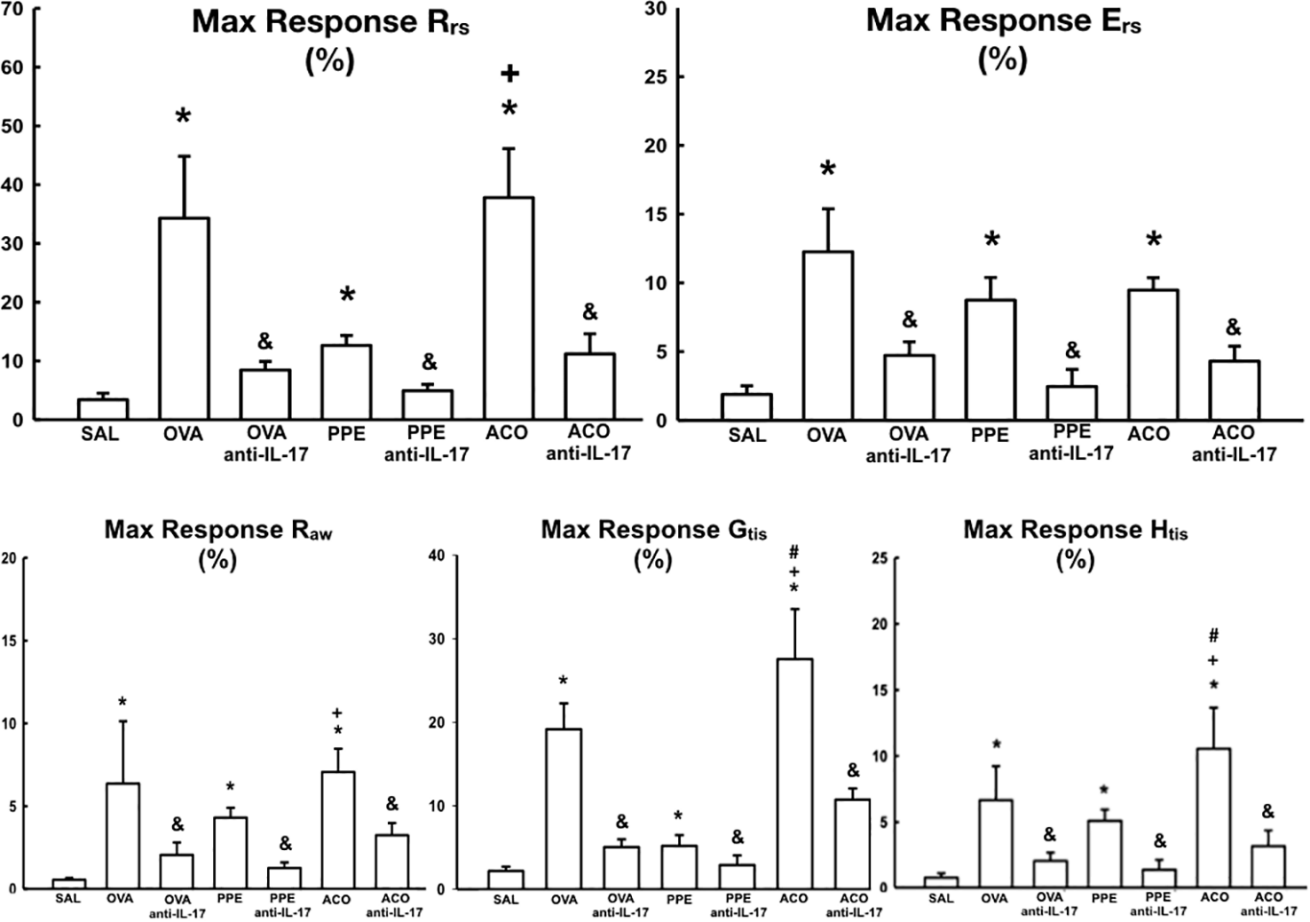
Results of airway hyperresponsiveness in relation to respiratory mechanics parameters (R_rs_, E_rs_, R_aw_, G_tis_, and H_tis_). The mean ± standard error of the maximum response after methacholine challenge for all experimental groups. Values are presented as percentages. Resistance (Rrs); Elastance (Ers); Resistance of larger airways (R_aw_); Resistance of smaller airways or tissues (G_tis_) and elastance of lung tissues (H_tis_). *p < 0.05, compared to the SAL group; ^+^p < 0.05 comparado ao grupo PPE; ^#^p < 0.05 comparado ao grupo OVA ^&^p < 0.05, compared to the OVA, PPE, and ACO groups.

The respiratory mechanics in the animals after challenge with methacholine in the seven experimental groups are presented in **Figure 3**. Compared to the SAL control group, the OVA, PPE, and ACO groups showed increased resistance (R_rs_), elastance (E_rs_), resistance of larger airways (R_aw_), resistance of smaller airways or tissues (G_tis_), and elastance of lung tissues (H_tis_) for all comparisons (p < 0.05). The maximum response of G_tis_ and H_tis_ in the ACO group was significantly higher compared to the OVA group (p < 0.05), for all comparisons. Similarly, we observed that the maximum response of R_rs_, R_aw_, G_tis_, and H_tis_ in the ACO group was higher than in the PPE group (p < 0.05), for all comparisons. There was a reduction in R_rs_, E_rs_, R_aw_, G_tis_, and H_tis_ in the OVA anti-IL-17, PPE anti-IL-17, and ACO anti-IL-17 groups compared to the OVA, PPE, and ACO groups, respectively (p < 0.05), for all comparisons.

### Mean alveolar diameter

**Figure 4:**
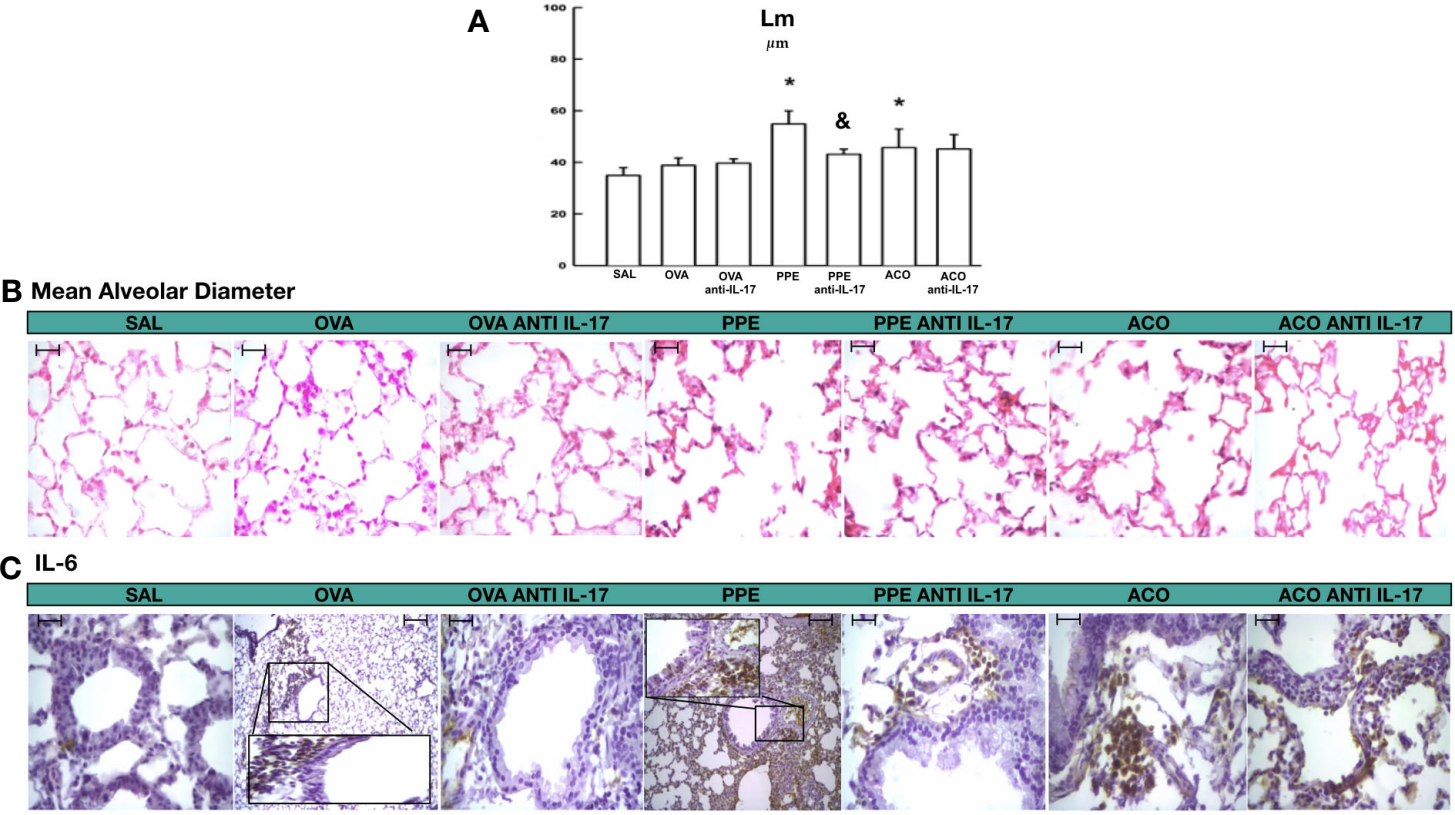
Results of mean alveolar diameter and qualitative analysis for IL-6. **(A)** Mean ± standard error of LM evaluation for all experimental groups. Values are presented in micrometers. *p < 0.05, compared to the SAL group; ^&^p < 0.05, compared to the PPE group. **(B)** Photomicrograph panel showing LM in HE staining **(C)** and IL-6 in the airway. All figures are presented at a magnification of 400× in the OVA and PPE groups, with scale bars = 40 µm.

Mean alveolar diameter is shown in **Figure 4A**. There was an increase in the mean alveolar diameter in the PPE and ACO groups compared to the SAL control group (p < 0.05). There was no difference between the OVA and PPE groups compared to the ACO group. Anti-IL-17 treatment attenuated this effect only in the PPE-treated group compared to the PPE group (p < 0.05). There was no difference among the other groups. In **Figures 4B, C**, photomicrographs illustrate the characteristics of the average alveolar diameter and IL-6 in animals across different experimental groups. An increase in the average alveolar diameter was observed in the PPE and ACO groups compared to the SAL group. Animals treated with anti-IL-17 (PPE anti-IL-17) exhibited a decrease in the average alveolar diameter compared to the PPE group. For IL-6, we observed an increase in the OVA, PPE, and ACO groups. Treatment with anti-IL-17, however, reduced the cytokines in the treated groups.

### Cells counts in bronchoalveolar lavage fluid

**Figure 5:**
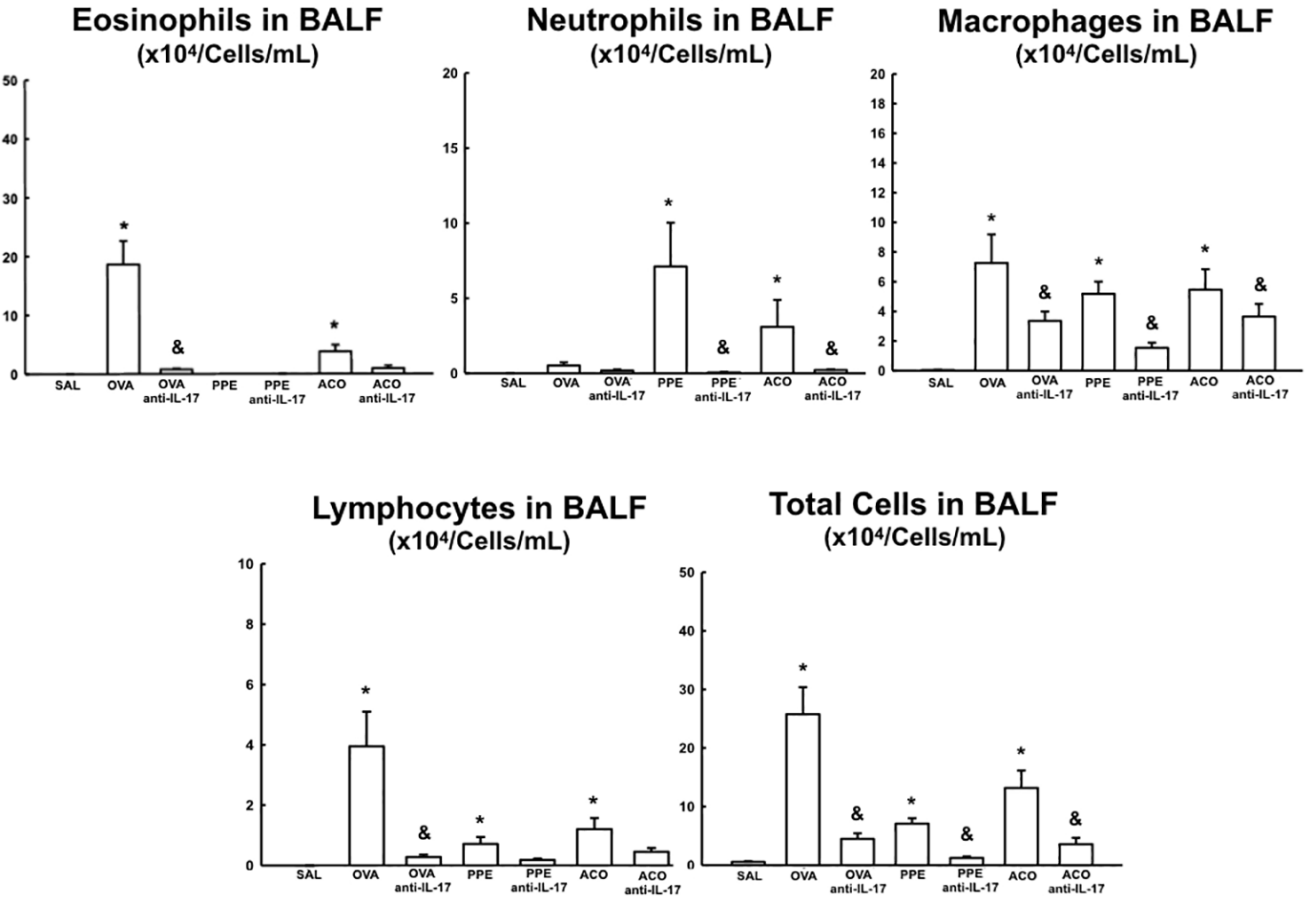
Results of eosinophils, neutrophils, macrophages, lymphocytes, and total cells in Bronchoalveolar Lavage Fluid (BALF). Mean ± standard error of BALF assessment for all experimental groups. Values are presented in x10^4^ cells/mL. *p < 0.05, compared to the SAL group; ^&^p < 0.05, compared to the OVA, PPE, and ACO groups.

The total cell count and differential count for eosinophils, neutrophils, macrophages, and lymphocytes in the bronchoalveolar lavage fluid are shown in **Figure 5**. There was an increase in the number of macrophages, lymphocytes, and total cells in the OVA, PPE, and ACO groups compared to the SAL group (p < 0.05). Relative to controls, eosinophils increased in the OVA but not in the PPE and ACO groups, while neutrophils increased in the PPE and ACO groups but not in the OVA group. We did not observe a significant increase in total and differential cell counts in the ACO group compared to the OVA and PPE groups. The animals in the OVA anti-IL-17, PPE anti-IL-17, and ACO anti-IL-17 groups showed attenuation in macrophage numbers and total cell count compared to the OVA, PPE, and ACO groups, respectively (p < 0.05), for all comparisons. Eosinophil and lymphocyte counts were attenuated only in the OVA anti-IL-17 group compared to the OVA group (p < 0.05). Neutrophils were reduced in the PPE anti-IL-17 and ACO anti-IL-17 groups compared to the PPE and ACO groups, respectively.

### Cell-associated inflammatory cytokines

**Figure 6:**
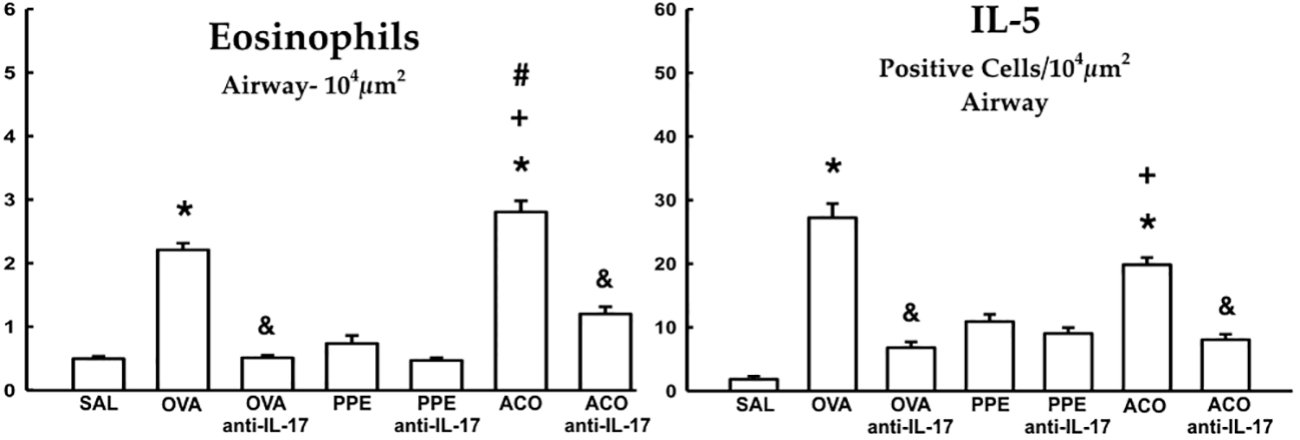
Results of positive cell expression assessed by hematoxylin and eosin (HE) staining for eosinophils and immunohistochemistry for IL-5 in the airways. Mean ± standard error of eosinophil and IL-5 assessments for all experimental groups. Values are presented in cells/10^4^ µm^2^. *p < 0.05, compared to the SAL group; ^+^p < 0.05 comparado ao grupo PPE; ^#^p < 0.05 comparado ao grupo OVA ^&^p < 0.05, compared to the OVA and ACO groups.

**Figure 7:**
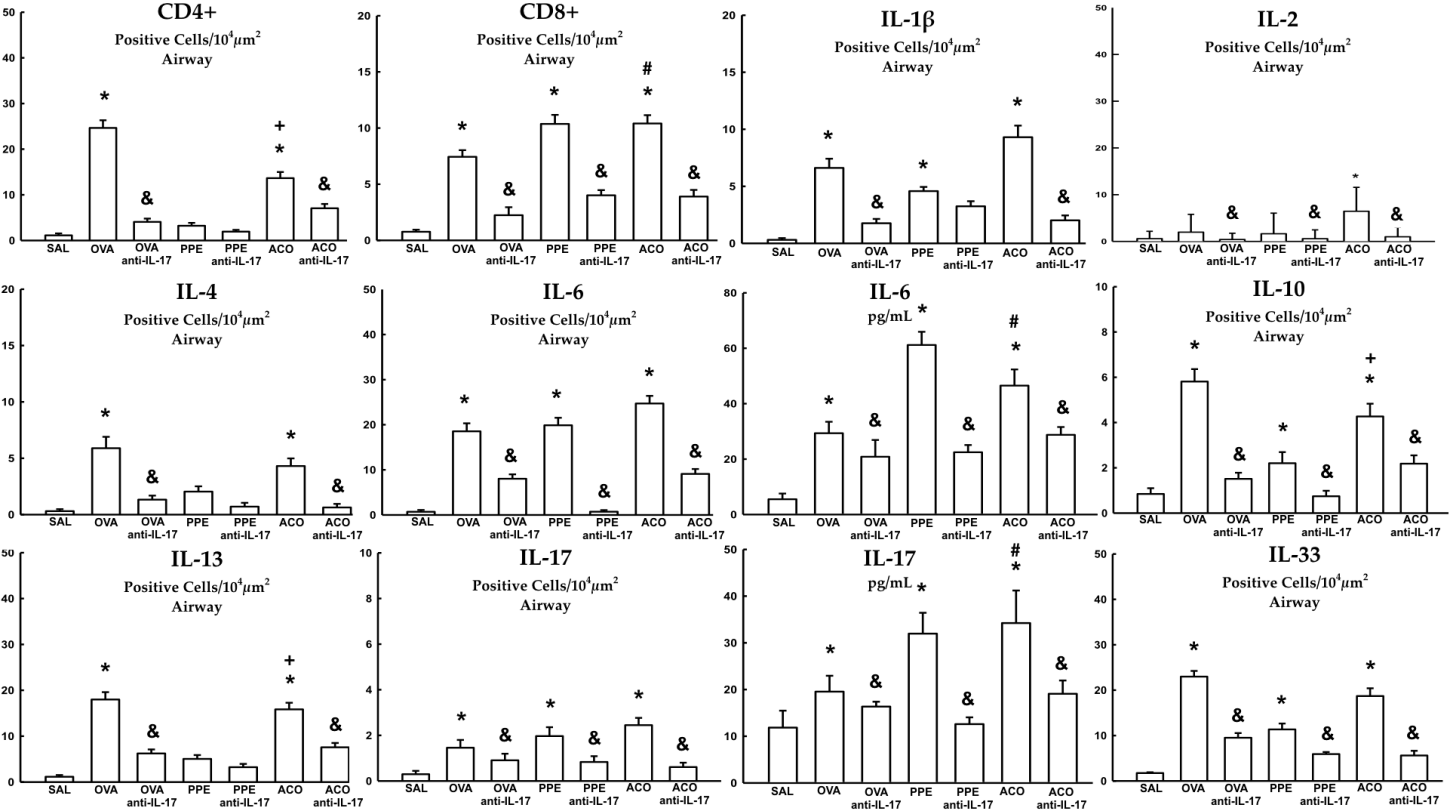
Results of positive cell expression assessed by immunohistochemistry for cell-associated cytokine production in the airway, and levels of IL-6 and IL-17 measured by ELISA. Mean ± standard error of CD4+, CD8+, IL-1β, IL-2, IL-4, IL-6, IL-10, IL-13, IL-17, and IL-33 assessment for all experimental groups. Values are presented in positive cells/10^4^ µm^2^ and pg/mL for IL-6 and IL-17. *p < 0.05, compared to the SAL group; ^+^p < 0.05 comparado ao grupo PPE; ^#^p < 0.05 comparado ao grupo OVA ^&^p < 0.05, compared to the OVA, PPE, and ACO groups.

**Figure 8:**
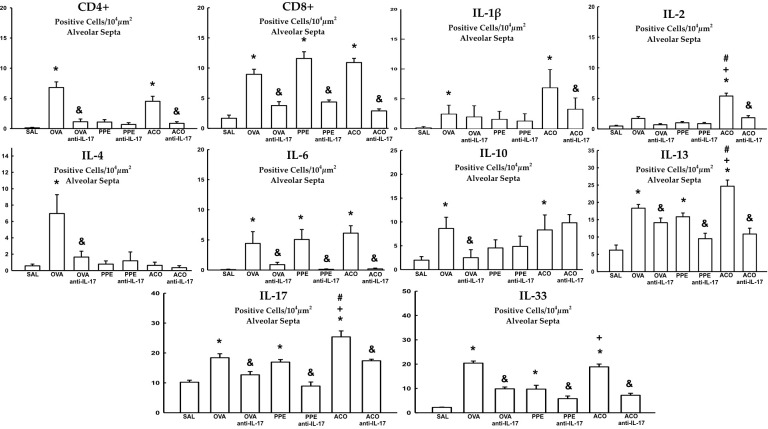
Results of positive cell expression assessed by immunohistochemistry for cell-associated cytokine production in the alveolar septa. Mean ± standard error of CD4+, CD8+, IL-1β, IL-2, IL-4, IL-6, IL-10, IL-13, IL-17, and IL-33 assessment for all experimental groups. Values are presented in positive cells/10^4^ µm^2^. *p < 0.05, compared to the SAL group; ^+^p < 0.05 comparado ao grupo PPE; ^#^p < 0.05 comparado ao grupo OVA ^&^p < 0.05, compared to the OVA, PPE, and ACO groups.

Cell-associated cytokine production for eosinophils, CD4+, CD8+, IL-1β, IL-2, IL-4, IL-5, IL-6, IL-10, IL-13, IL-17, and IL-33 in the airway, as well as IL-6 and IL-17 levels for all experimental groups, are presented in **Figures 6**, **7**, while alveolar septa data is shown in **Figure 8**. A notable elevation in the production of cell-associated cytokines was detected in the lung tissue, particularly for eosinophils and IL-5 within the airways. Furthermore, IL-1β, IL-6, IL-10, IL-13, IL-17, and IL-33 exhibited increased levels, both in the airways and in the alveolar septa, in the OVA and ACO groups as compared to the SAL group (p < 0.05). IL-2-expressing cells did not increase in the OVA and PPE groups compared with the SAL group. In the PPE group, airway IL-4 and IL-13 cells and alveolar septal IL-1β, IL-4, and IL-10 cells did not differ compared to the SAL group. In the ACO group, the IL-4 cell count was not significantly different compared to SAL controls. We observed an increase in cell-associated cytokine production for eosinophils, CD4+, IL-5, IL-10, and IL-13 in the airway, and for IL-2, IL-13, IL-17, and IL-33 in the alveolar septa of the ACO group compared to the PPE group (p < 0.05). Similarly, we identified a similar pattern in the airway for eosinophils, IL-2, IL-13, and IL-17, and IL-2, IL-13, and IL-17 in the alveolar septum of the ACO group when compared to the OVA group (p < 0.05). The animals that received the anti-IL-17 neutralizing antibody (OVA anti-IL-17, PPE anti-IL-17, and ACO anti-IL-17) generally exhibited a reduction in cell-associated cytokine production in both the airways and alveolar septa when compared to the OVA, PPE, and ACO groups, respectively (p < 0.05) with some exceptions. When comparing the PPE and PPE anti-IL-17 groups, there was no difference in the cells expressing eosinophils, IL-1β, IL-4, and IL-13 in the airway and IL-1β, IL-2, and IL-4 in the alveolar septum. There was no attenuation in alveolar septal IL-1β cells in the anti-IL-17 OVA group and IL-4 cells in the ACO anti-IL-17 group.

### Extracellular matrix remodeling

**Figure 9:**
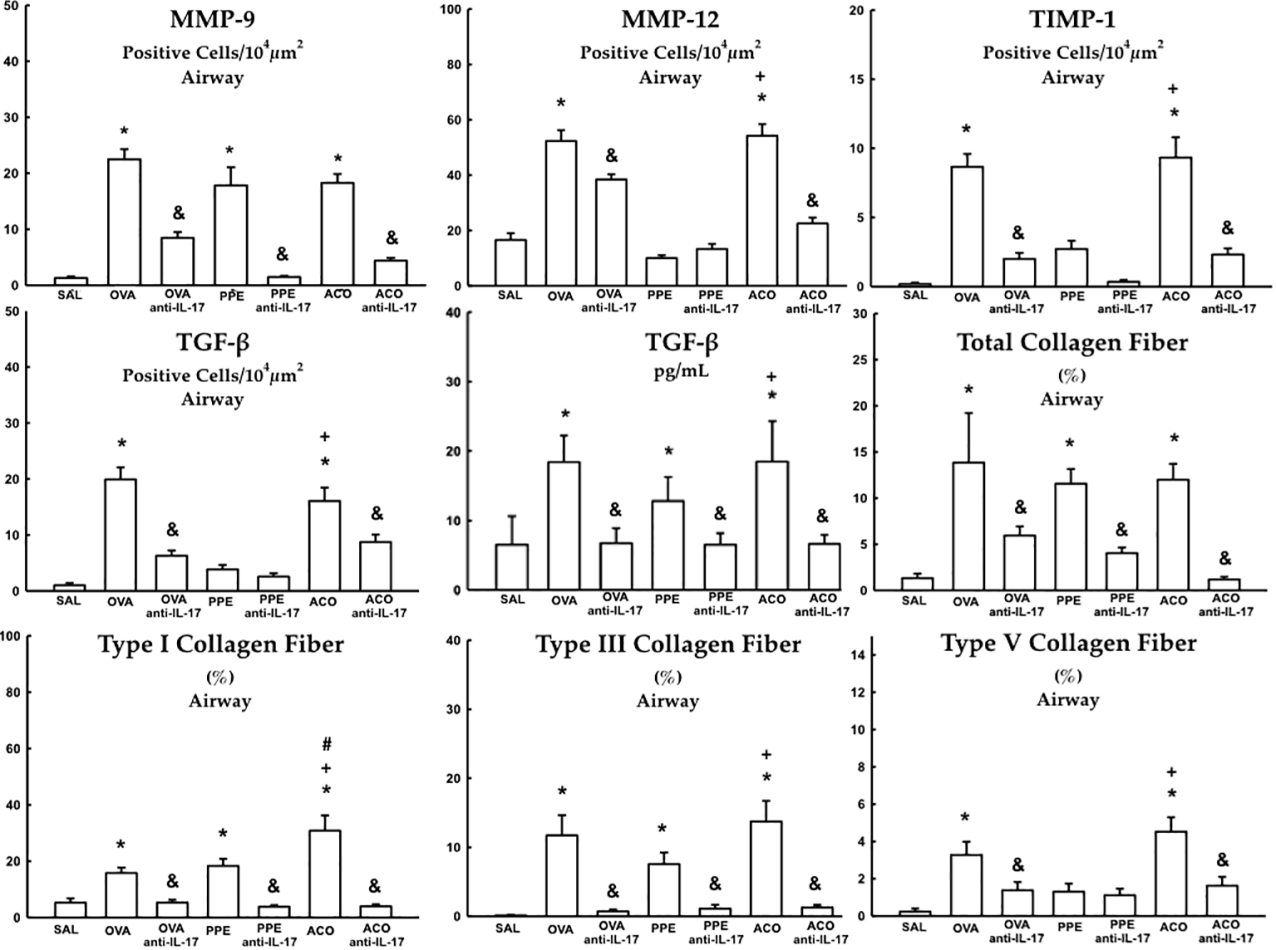
Results of positive cell expression assessed by immunohistochemistry for extracellular matrix remodeling in the airway and levels of TGF- β measured by ELISA. Mean ± standard error of MMP-9, MMP-12, TIMP-1, TGF-β and total collagen fiber content, types I, III, and V assessment for all experimental groups. Values are presented in positive cells/10^4^ µm^2^, % and pg/mL for TGF-β. *p < 0.05, compared to the SAL group; ^+^p < 0.05 comparado ao grupo PPE; ^#^p < 0.05 comparado ao grupo OVA ^&^p < 0.05, compared to the OVA, PPE, and ACO groups.

Cells positive for MMP-9, MMP-12, TIMP-1, TGF-β, and content of total collagen fibers, type I, III, and V collagen fibers in the airway, as well as TGF-β levels, are presented in **Figure 9**. Meanwhile, data for the alveolar septa is shown in **Table 1**, respectively. These markers of extracellular matrix remodeling were elevated in the OVA, PPE, and ACO groups compared to the SAL group (p < 0.05), except for airway MMP-12, TIMP-1, TGF-β, and collagen fibers type V and alveolar septal total collagen in the PPE group. There was an increase in the airway for MMP-12, TIMP-1, TGF-ß, collagen fibers type I, III, and V, as well as TGF-ß levels. In the alveolar septum, an increase in TIMP-1 and collagen type V was observed in the ACO group compared to the PPE group (p < 0.05). We observed a similar trend in the airway in the ACO group regarding collagen type I and in the alveolar septum for TGF-ß when compared to the OVA group (p < 0.05). Animals that received anti-IL-17 neutralizing antibody (OVA anti-IL-17, PPE anti-IL-17, and ACO anti-IL-17) showed attenuation of these markers when compared to the OVA, PPE, and ACO groups, respectively (p < 0.05), for most comparisons. In the PPE anti-IL-17 and PPE groups, MMP-12, TIMP-1, and TGF-β, and type V collagen fibers in the airway and total collagen and TIMP-1 in the alveolar septa showed no difference. MMP-12 and total collagen fiber did not differ in the alveolar septa of OVA anti-IL-17 and OVA groups. Total collagen fiber content was unchanged following anti-IL-17 treatment in the ACO group.

**Table 1:** Absolute values of remodeling markers in the alveolar septa.

PULMONARY REMODELING MARKERS	SAL	OVA	OVAANTI-IL-17	PPE	PPEANTI-IL-17	ACO	ACOANTI-IL-17
**MMP-9** **(Positive Cells/10^4^µm^2^)**	8.11 ± 1.72	20.89 ± 1.48*	11.41 ± 1.03&	11.65 ± 1.61*	7.20 ± 0.68&	15.79 ± 1.82*	7.93 ± 1.13&
**MMP-12** **(Positive Cells/10^4^µm^2^)**	13.41 ± 2.98	23.94 ± 0.94*	18.99 ± 0.92	21.13 ± 1.80*	15.13 ± 1.85&	23.72 ± 1.64*	14.61 ± 1.86&
**TIMP-1** **(Positive Cells/10^4^µm^2^)**	0.17 ± 0.17	11.25 ± 3.56*	3.84 ± 1.82&	2.45 ± 1.39*	1.48 ± 0.80	10.08 ± 3.84*+	4.39 ± 1.96&
**TGF-β** **(Positive Cells/10^4^µm^2^)**	0.16 ± 0.10	5.93 ± 2.34*	0.89 ± 0.57&	9.57 ± 2.72*	0.50 ± 0.17&	12.35 ± 2.16*#	1.78 ± 0.93&
**Total Collagen Fiber (%)**	0.01 ± 0.08	1.32 ± 3.97*	0.55 ± 2.25	0.20 ± 1.52	0.19 ± 1.49	1.23 ± 3.05*	0.02 ± 0.1
**Type I Collagen Fiber (%)**	2.07 ± 0.43	9.98 ± 1.99*	4.1 ± 0.50&	9.08 ± 1.25*	2.60 ± 0.46&	14.02 ± 1.51*	3.53 ± 0.57&
**Type III Collagen Fiber (%)**	0.35 ± 0.05	9.42 ± 1.54*	1.49 ± 0.35&	3.93 ± 0.87*	0.12 ± 0.04&	8.28 ± 1.23*	2.54 ± 0.40&
**Type V Collagen Fiber (%)**	1.84 ± 0.38	7.65 ± 1.44*	3.77 ± 0.68&	3.47 ± 0.81	2.07 ± 0.38&	9.01 ± 0.63*+	1.77 ± 0.26&

Remodeling markers for MMP-9, MMP-12, TIMP-1 and TGF-ß are expressed as positive cells/10^4^µm^2^. Total collagen fibers and subtypes I, III and V are presented as volume fraction expressed as percentages of area (%). Mean ± standard error of remodeling markers assessment for all experimental groups. Values are presented as absolutes. *p < 0.05, compared to the SAL group; + p < 0.05 comparado ao grupo PPE; # p < 0.05 comparado ao grupo OVA; &p < 0.05, compared to the OVA, PPE, and ACO groups.

### Exhaled nitric oxide and oxidative stress

The eNO concentration, iNOS-positive cell count, and 8-iso-PGF-2α content in the airway and alveolar septa are shown in **Table 2**. There was an increase in eNO concentration and iNOS+ cells in the airway and alveolar of animals in the OVA, PPE, and ACO groups compared to the SAL group (p < 0.05). Compared to controls, elevated 8-iso-PGF-2α were observed in all groups except in the PPE group. In the airway, we observed a significant increase in the content of 8-iso-PGF-2α in the ACO group compared to the PPE group (p < 0.05), as well as in the number of iNOS-positive cells in the alveolar septum of the ACO group compared to the OVA group (p < 0.05). Anti-IL-17 neutralizing antibody attenuated iNO-positive cells in the treated OVA, PPE, and ACO groups, compared to their respective controls (p < 0.05). Elevated eNO concentration and 8-iso-PGF-2α content were observed in the OVA and ACO anti-IL-groups but not in the PPE anti-IL-17 group.

**Table 2:** Absolute values of remodeling markers in the airway and alveolar septa.

OXIDATIVE STRESS MARKERS	SAL	OVA	OVA ANTI-IL-17	PPE	PPE ANTI-IL-17	ACO	ACO ANTI-IL-17
**8-iso-PGF-2**α **(%) Airway**	0.19 ± 0.06	5.49 ± 1.36*	0.53 ± 0.12&	1.70 ± 0.89	0.86 ± 0.26	8.27 ± 2.38*+	4.56 ± 1.08&
**8-iso-PGF-2**α**(%) Alveolar Septa**	0.42 ± 0.02	2.20 ± 1.03*	1.67 ± 0.16	2.19 ± 0.42*	0.51 ± 0.01&	2.64 ± 0.61*	0.92 ± 0.04&
**iNOS (Positive Cells/10^4^µm^2^) Airway**	4.07 ± 0.74	30.01 ± 1.81	15.37 ± 1.45&	23.11 ± 0.56*	11.670 ± 1.10&	25.12 ± 2.35*	12.91 ± 1.21&
**iNOS (Positive Cells/10^4^µm^2^) Alveolar Septa**	11.49 ± 2.72	18.44 ± 0.94*	12.05 ± 1.33&	21.47 ± 3.23*	13.75 ± 0.74&	26.71 ± 2.07*#	20.18 ± 2.83&
**Nitric Oxide Exhaled (pph)**	5.30 ± 2.49	24.17 ± 4.30*	9.75 ± 1.14&	9.61 ± 2.19	8.74 ± 1.23	13.73 ± 4.42*	9.42 ± 2.50

Oxidative stress for 8-iso-PGF-2α, iNOS in airway and alveolar septa and Nitric oxide exladed. Mean ± standard error of Oxidative stress assessment for all experimental groups. Values are presented as absolutes. *p < 0.05, compared to the SAL group; + p < 0.05 comparado ao grupo PPE; # p < 0.05 comparado ao grupo OVA; &p < 0.05, compared to the OVA, PPE, and ACO groups.

### Signaling pathways

**Figure 10:**
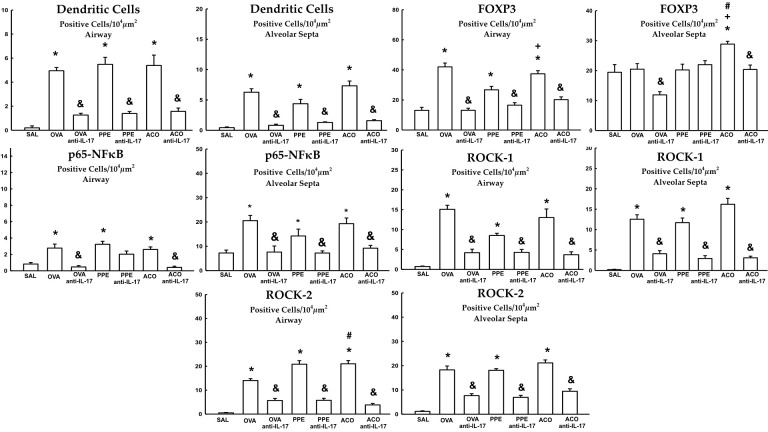
Results of positive cell expression assessed by immunohistochemistry for signaling pathways in the airway and alveolar septa. Mean ± standard error of dendritic cells, FOXP3, p65-NFκB, ROCK-1 and ROCK-2 assessment for all experimental groups. Values are presented in positive cells/10^4^ µm^2^ *p < 0.05, compared to the SAL group; ^+^p < 0.05 comparado ao grupo PPE; ^#^p < 0.05 comparado ao grupo OVA ^&^p < 0.05, compared to the OVA, PPE, and ACO groups.

Quantification of dendritic cells, FOXP3+, p65-NFκB, ROCK-1, and ROCK-2 in the airway and alveolar septa are shown in **Figures 10**. Animals in the OVA, PPE, and ACO groups showed an increased number of expressing dendritic cells, FOXP3, p65-NFκB, ROCK-1, and ROCK-2 cells in the airway and alveolar septa compared to the SAL group (p < 0.05), except FOXP3 in the OVA and PPE groups in the alveolar septa. We observed a significant increase in FOXP3 production in both the airway and alveolar septum within the ACO group when compared to the PPE group (p < 0.05). Similarly, within the alveolar septum, there was a notable increase in FOXP3 and ROCK-2 production in the ACO group compared to the OVA group (p < 0.05).Animals that received anti-IL-17 neutralizing antibody (OVA anti-IL-17, PPE anti-IL-17, and ACO anti-IL-17) showed attenuation in the markers for intracellular signaling in the airway and in the alveolar septa when compared to the OVA, PPE, and ACO groups, respectively (p < 0.05), for all comparisons, except for FOXP3 in the alveolar septa and p65-NFκB in the airway of animals in the PPE anti-IL-17 group.

### Qualitative analysis of the effects of anti-IL-17 on the airway

**Figure 11:**
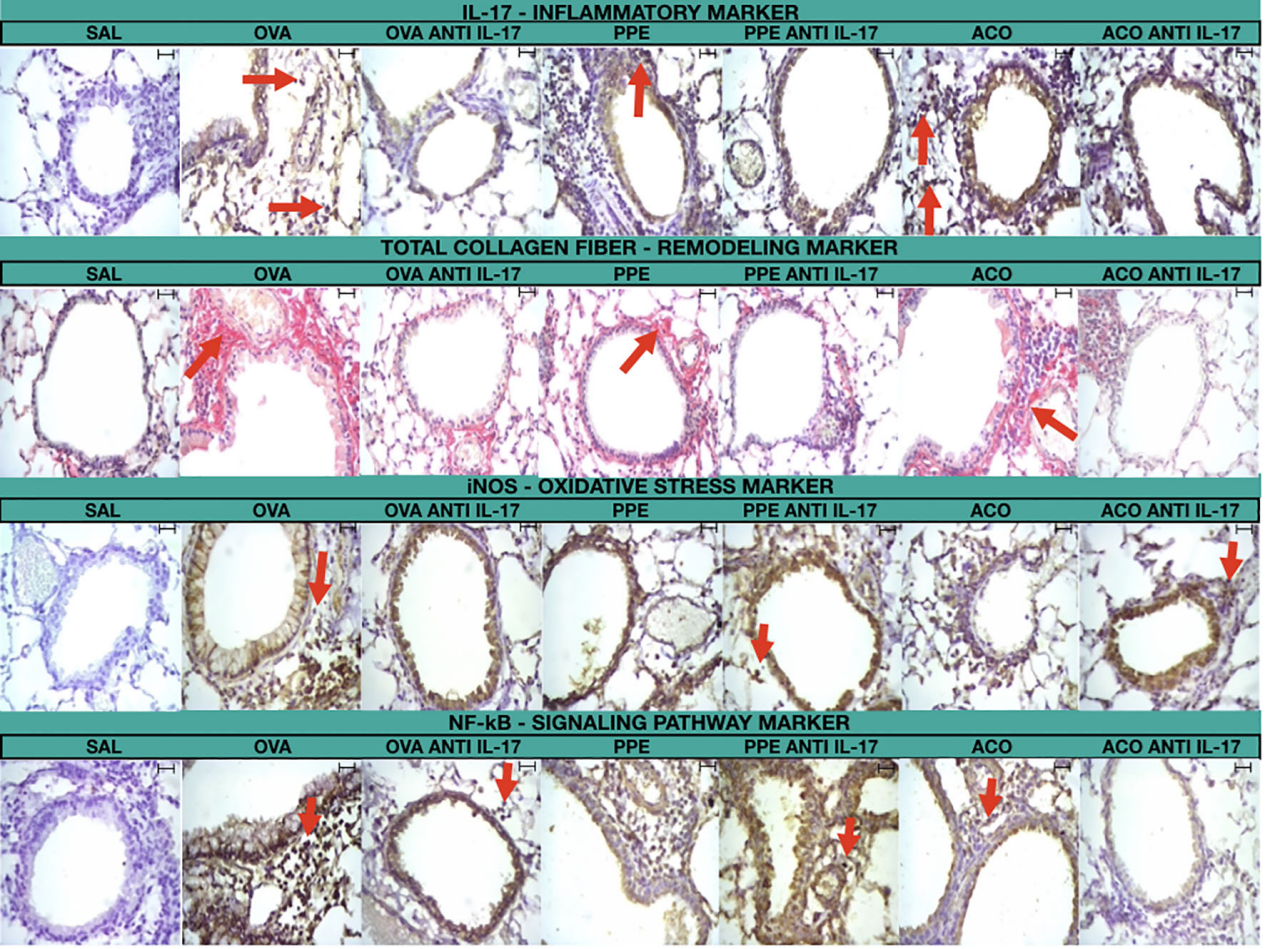
Photomicrograph of immunohistochemical staining of airway walls to detect IL-17, total collagen fiber, iNOS, and NF-κB (400× magnification)/Scale bar = 40 μm.

**Figure 11** includes photomicrographs that illustrate the inflammatory features, remodeling, oxidative stress markers, and signaling pathway markers in the lungs of animals across experimental groups. A significant increase in cell-associated cytokine production was observed in the lung tissue of animals exposed to OVA, PPE, and ACO, in comparison to the SAL group. Animals treated with anti-IL-17 showed attenuation in all groups. Comparative results between ACO and asthma and/or COPD in the form of illustrations can be found in the **Supplementary Material**.

## Discussion

In the present study, we evaluated the effects of IL-17 inhibition using an anti-IL-17 monoclonal antibody in an experimental model of asthma–COPD overlap (ACO). We analyzed the responses of hyperresponsiveness to methacoline, identify of cell-associated cytokine production in lung tissue, markes of extracellular matrix remodeling and, signaling pathway markers, as well as oxidative stress markers. Following anti-IL-17 treatment, animals sensitized with ovalbumin, received elastase, or both showed attenuation in the hyperresponsiveness of the large and small airways after the challenge with methacholine and lung tissue elastance. These results were associated with a reduction in the number of eosinophils, neutrophils, lymphocytes, and macrophages in the bronchoalveolar lavage fluid and decreased of cell-associated cytokine production in lung tissue, such as (IL-1β, IL-2, IL-4, IL-5, IL-6, IL-13, and IL-17). CD4+ cells were fewer in the OVA and ACO anti-IL-17 groups but not in the PPE anti-IL-17 group, which also showed no reduction in IL-1, IL-5, and IL-13. Treatment with anti-IL-17 reduced anti-inflammatory cytokines and regulatory T cells (IL-10 and FOXP3) and induced control of lung remodeling, as evidenced by a reduction in the total collagen fibers and their subtypes (I, III, and V) and the number of cells positive for TIMP-1, MMP-9, MMP-12, and TGF-β. IL-17 inhibition also reduced oxidative stress markers assessed by the expression of iNOS-positive cells, 8-iso-PGF-2α, and exhaled NO. These findings were seen in both the airway and alveolar septa.

The use of monoclonal antibodies for the treatment of specific asthma phenotypes has achieved greater control of the disease. However, about 5 to 10% of patients with asthma do not respond to high doses of oral or inhaled corticosteroids and are not suitable candidates for antibody therapies due to different inflammatory patterns (30). Neutrophilic predominance and elevated IL-17 are markers that determine nonresponse to conventional asthma therapies (31). In comparison, COPD, which follows a neutrophilic inflammatory pattern, requires further elucidation of its pathophysiological mechanisms and new treatment options to control the disease.

Airway hyperresponsiveness (AHR) is a hallmark of asthma, COPD, and ACO (32). Studies suggest that IL-4, IL-13, and IL-17A mediate increased smooth muscle contractile force after allergen challenge (33, 34). We observed AHR in the untreated models of asthma, COPD, and ACO, which was most prominent in the OVA group. Importantly, anti-IL-17 treatment attenuated small and large airway hyperresponsiveness in the different treated groups. It is worth noting that we observed a significant reduction in the maximum bronchoconstrictor response with anti-IL-17 treatment, as we can see: OVA anti-IL-17, Rrs (-75.38%), Raw (-67.71%), Gtis (-73.66%). ACO anti-IL-17, Rrs (-70.38%), Raw (-54.04%), and Gtis (-61.06%).

In a study by Ikeda et al. (23), induction of ACO using egg albumin and PPE in mice resulted in increased AHR, static compliance, inflammatory cell infiltration, cytokine levels, and mean linear intercept. Treatment of these animals with montelukast, an anti-leukotriene, reduced AHR, static compliance, eosinophilic and neutrophilic infiltration of the airways, and progression to emphysema. We had similar observations in our experimental model of ACO, which showed lung damage and histopathological changes. When we treated the animals with an anti-IL-17 antibody, we observed control and reduction of AHR, as well as lower R_aw_, E_rs_, total cell counts in the BALF, eosinophilic and neutrophilic inflammation, and mean linear intercept, which is a marker of pulmonary emphysema.

Bronchoalveolar lavage fluid is an efficient method to verify pulmonary inflammation (35). We observed a significant increase in neutrophils in the ACO group, as well as eosinophils in the OVA group. Eosinophil accumulation is a prominent feature of the allergic reactions that occur in asthma (36) and correlates with increased bronchial hyperresponsiveness. Although some regulatory mediators are involved in the recruitment of eosinophils to the lung, IL-5 has potent effects on eosinophils and is also involved in AHR. It promotes eosinophil growth, differentiation, proliferation, and chemotaxis (37, 38). A study in patients with COPD showed that septal eosinophils are positively correlated with hyperresponsiveness and sputum eosinophils are inversely correlated with FEV_1_ (39). Our data revealed increased R_aw_, BALF eosinophils, and IL-5 in the OVA and ACO groups. Treatment with anti-IL-17 was able to attenuate eosinophilia and IL-15 and, consequently, led to improvement in AHR.

Interleukins play an important role in the inflammatory processes in asthma and COPD (40). As a first line of defense, the innate immune system provides a nonspecific yet rapid response against exogenous agents to prevent damage. When this first line of defense is not sufficient, activation of the adaptive immune response occurs. This process occurs in chronic allergic inflammation and COPD (41).

In evaluating the activation of both pathways, we observed an increase cell-associated cytokine production for eosinophils, CD4+, IL-1β, IL-2, IL-4, IL-5, IL-6, IL-10, IL-13, and IL-17 in the group sensitized with OVA and animals which received the combination of elastase + OVA. However, we did not notice an increase in CD4+ cells, IL-2, IL-4, and IL-10 in the PPE group.

In COPD, an increase in activated dendritic cells and CD4+ and CD8+ T cells can be observed in the peripheral airways. King et al. (42) observed that both CD4+ and CD8+ T cells from individuals with COPD produce high levels of TNF-α, which contributes to the intensification of inflammation and tissue damage (43).

Naïve CD4+ T cells can differentiate into different subtypes (Th1, Th2, Th17, or Treg) depending on the cytokines present in the microenvironment (44). We observed an increase in interleukins IL-4, IL-5, and IL-13 in the OVA group, indicating the proliferation of effector cells in the Th2 response. In both the OVA and ACO groups, TGF-beta and IL-6 were increased, consistent with activation of Th17 (45).

Recently, Fukuzaki et al. (18) demonstrated that animals that received intratracheal elastase developed pulmonary emphysema and had a greater number of IL-17 cells. In this work, they used anti-IL-17 therapy prophylactically and therapeutically. Their results are in line with our findings in the ACO group, with anti-IL-17-treated animals showing lower IL-17 and alveolar damage.

Chen et al. (46) reported that IL-17^–/–^ mice do not develop emphysema even after six months of exposure to cigarette smoke, reinforcing the pathogenic role of this cytokine. Furthermore, Kurimoto et al. (47) observed reductions in neutrophil recruitment and emphysematous changes in the lungs of IL-17^–/–^ mice subjected to elastase instillation.

Our immunohistochemistry results showed an increase in IL-10 and TGF-β in the airway and alveolar septa of animals in the OVA, PPE, and ACO groups, except for TGF-β in the PPE group. Forkhead box P3 (FOXP3) was increased in the airway in the OVA, PPE, and ACO groups and in the alveolar septum in the ACO group. However, in the ELISA assay, there was a reduction of IL-10 in the PPE and ACO groups compared to the SAL group and an increase in TGF-β in the OVA, PPE, and ACO groups.

Treg cells control the inflammatory process by suppressing the activity of effector T cells via the secretion of anti-inflammatory cytokines, such as IL-10 and TGF-β (48). Lee et al. (49) were one of the first authors to propose that a reduction in Treg cell values was associated with the progression of COPD. They also detected a reduction in IL-10 expression in tissues of patients with COPD.

Although our model was elastase-induced emphysema, we found an increase in IL-10 in the airway and in the alveolar septum compared to the control. Moreira et al. (50) used a model of emphysema that was induced by elastase and exposure to ultrafine particles of pollutants exhausted by diesel combustion. Their results showed an increase in the number of IL-10-positive cells compared to the control.

FOXP3 induces Treg cell differentiation and expansion and serves as a specific marker for this process. Mice with Foxp3 deficiency (−/−) and patients with mutations or deletions in the foxp3 gene show interrupted Treg cell development, leading to fatal autoimmune and inflammatory diseases (51, 52). Once Tregs are activated, they express high levels of TGF-β and IL-10 (53).

In models of chronic allergic inflammation induced by ovalbumin, there is an increase in the expression of FOXP3, TGF-β, and IL-10 and treatment with anti-IL-17 was able to attenuate these markers (17). These results corroborate our findings. We hypothesize that the attenuation of Tregs is due to IL-17 inhibition-induced control of the inflammatory process.

We observed an increase in MMP-9, MMP-12, TIMP-1, TGF-β, and the content of total collagen fibers, type I, III, and V collagen fibers in the airway and alveolar septum in animals sensitized with ovalbumin (OVA group) and in animals that received the combination of elastase + OVA (ACO group). However, we did not observe an increase in MMP-12, TIMP-1, TGF-β, and Type V Collagen in the airway in the PPE group; the same was observed for total collagen fiber in the alveolar septum.

Corroborating our findings (6), in an experimental model of asthma induced by lipopolysaccharide (LPS), there was an increase in the number of cells positive for MMP-9, MMP-12, TIMP-1, and TGF-β, as well as an increase in the volume fraction of collagen fibers I and III, decorin, actin, biglycan, lumican, and fibronectin in the alveolar septum. These animals were also treated with an anti-IL-17 antibody and showed decreased lung inflammation, edema, and airway remodeling compared to untreated animals. Another study by our group used the same experimental model to show that these alterations are also present in pulmonary vessels, suggesting the role of extracellular matrix components in lung remodeling (16).

The experimental models of COPD that best represent structural lung changes are cigarette smoke (CS) exposure or elastase administration (54). We decided to use elastase because previous studies showed an increase in MMP-9, MMP-12, TGF-β, and type I and III collagens in this model. Furthermore, it only requires a short time to induce drastic structural changes, compared to CS induction (55, 56).

MMP-12 is the most frequently described metalloproteinase in animal models of COPD and, together with TGF-β, acts by modulating the amounts of elastic and collagen fibers (57).

Structural changes are observed mainly in the alveolar septa, where alveolar enlargement is observed. This reflects the destruction of the alveolar wall and the presence of fragmented elastic and collagen fibers (18). We noticed that there was an increase in the LM in the PPE and ACO groups and treatment with anti-IL-17 ameliorated alveolar enlargement. This improvement may be because anti-IL-17 attenuated TGF-β signaling and, consequently, downregulated total collagen and its subtypes.

Increased oxidative stress in the lungs of patients with asthma, COPD, and ACO is well-established. Oxidative stress is a major driver of chronic inflammation, disease progression, and exacerbations, which are worse in patients with ACO (13, 32, 58).

Exhaled nitric oxide (NO) and 8-iso-PGF2α are the main biomarkers of oxidative stress, especially in asthma (59, 60). We showed an increase in exhaled NO and 8-iso-PGF2α in the OVA, PPE, and ACO groups, especially in the OVA group. Likewise, the number of iNOS-positive cells was increased in the three untreated groups, both in the airway and alveolar septa.

Prado et al. (61) demonstrated that iNOS inhibition reduced MMP-9, TIMP-1, and TGF-β levels in the airways during chronic allergic inflammation, in addition to controlling oxidative stress. Recently Fukuzaki et al. (18) demonstrated that two isoforms of nitric oxide synthase, eNOS and iNOS, participate in oxidative stress in elastase-induced emphysema models. Anti-IL-17 treatment attenuated these markers and decreased oxidative stress. These data are consistent with our findings.

Another study showed that intranasal instillation of elastase in mice induced pulmonary emphysema and increased the expression of MMPs, NF-κB, and 8-iso-PGF-2α in the airways. Animals treated with flavanone showed a decrease in elastase-induced emphysema through the regulation of NF-κB, oxidative stress, and metalloproteinases (62).

Ween et al. (63) recently found the presence of lipid oxidation products in the airways of mice exposed to CS. Furthermore, lipid oxidation and 8-iso-PGF-2α levels are increased in the bronchoalveolar lavage fluid of smokers, COPD smokers, and former smokers with COPD.

The 8-iso-PGF-2α, an isomer of PGF2a, has contractile effects upon binding to thromboxane A2 receptors on airway smooth muscle (64). This agent causes increased lung resistance, indicating its role as a mediator of airflow limitation (65). Previous studies that used anti-IL-17 in models of chronic allergic inflammation showed that the inhibition of this cytokine modulated oxidative stress responses (6, 17, 18). These data corroborate our results in this study.

The transcription factor NF-κB is an important modulator of inflammation in the pathogenesis of lung diseases (66). Airway epithelial cells secrete NF-κB, pro-inflammatory cytokines, and chemokines, and regulate dendritic cells, the main antigen-presenting cells of the immune system (67). NF-κB signaling is triggered by ROS (68). Oxidative stress and ROS-induced cell injury can directly activate p38 and c-Jun N-terminal kinase, subsequently resulting in the activation of NF-κB (69).

According to Gagliardo et al. (70), the exaggerated activation of NF-κB perpetuates the production of inflammatory mediators in severe asthma. Several NF-κB downstream targets, such as IL-1β, monocyte chemotactic protein-1, TGF-β, and iNOS, are upregulated in inflammatory lung diseases (71). In a model of murine asthma, Pantano et al. (66) demonstrated that epithelial activation of NF-κB promoted neutrophilia and eosinophilia and increased levels of IL-17 and IL-4 (66, 70). Notably, activation of this pathway can also increase iNOS and arginase levels (72). Using an experimental model of LPS-induced emphysema, M. Chen et al. (73) showed increased levels of IL-6, IL-8, and TNF-α via activation of the NF-κB pathway.

In our study, we observed an increase in NF-κB signaling in the untreated animals in the OVA, PPE, and ACO groups. Treatment with anti-IL-17 was able to attenuate this pathologic response. We hypothesize that anti-IL-17 treatment controlled cell-associated cytokine production in lung tissue by modulating NF-κB.

Our study has some limitations. We did not use an ACO model exposed to CS, an experimental model that best mimics the etiology of COPD.

The use of an isotype-matching antibody is a common practice for control in most studies, and the absence of this type of control is a limitation in our study. However, it is important to note that implementing such a control incurs additional costs for research. On the other hand, in many studies, the control group is represented by the vehicle in which the test drug was dissolved, as is the case in our study, where the control group consists of the use of saline solution. Additionally, this approach is supported by other studies addressing allergic inflammation models or involving anti-IL-17 (16–18). In previous studies, we successfully used the anti-IL-17 antibody to reduce IL-17 levels in LPS-induced exacerbated asthma models, without observing non-specific effects (6). In our current study, we again observed a reduction in IL-17 levels, confirmed by ELISA and immunohistochemistry. Furthermore, the anti-IL-17 treatment significantly reduced various inflammatory markers, remodeling, oxidative stress, and signaling pathways, indicating a substantial, rather than marginal, treatment effect.

We used a monoclonal antibody targeting IL-17 in mice to explore this therapeutic approach for ACO. However, it is important to note that direct extrapolation of these findings to humans should be done with caution. Nevertheless, it is worth mentioning that several clinical trials are currently underway to test novel treatments for severe asthma, while COPD therapeutic options remain limited. Another limitation is that, for the identification of isoprostane in immunohistochemistry, we used the same method as the other antibodies in the blocking step. This involved the use of methanol, which potentially could have partially affected the detection of isoprostane.

However, this study has several strengths. We previously demonstrated the importance of the Th17 profile in chronic allergic inflammation. We have now decided to explore a model of ACO that has been little investigated in the literature. Although the emphysema induction was achieved using elastase, we demonstrated pathological changes consistent with COPD in a short period of time. It is essential to emphasize that the pathophysiology of ACO is not yet fully understood, limiting our ability to conduct trials that would better control patient symptoms and enhance their quality of life, and potentially even improve survival. In this regard, our asthma-COPD overlap model proves to be a valuable animal model due to its ability to identify similar changes in both diseases within a relatively short period of time. We observed the recruitment of inflammatory cells, increased airway hyperresponsiveness, as well as tissue remodeling with the recruitment of MMPs and collagen fibers. In addition to the presence of signaling markers, we also observed the potential perpetuation of inflammatory and oxidative stress markers. This allows us to investigate the cellular and molecular mechanisms believed to underlie the pathogenesis of asthma and COPD, contributing to functional changes that aggravate symptoms and worsen the condition.This demonstrates the importance of further exploration of this model. Our results support the importance of anti-IL17 therapy in attenuating and controlling inflammatory responses, remodeling, and activation of oxidative stress and NF-κB pathways in ACO. However, additional studies are warranted to reveal other pathways that may be involved in these responses.

## Data availability statement

The raw data supporting the conclusions of this article will be made available by the authors, without undue reservation.

## Ethics statement

The animal study was approved by Research Ethics Committee of the University of São Paulo School of Medicine (Protocol No. 1029/2018). The study was conducted in accordance with the local legislation and institutional requirements.

## Author contributions

LC: Data curation, Formal Analysis, Investigation, Methodology, Writing – original draft, Conceptualization, Validation. RR: Data curation, Formal Analysis, Investigation, Writing – review & editing. FD: Formal Analysis, Investigation, Methodology, Writing – review & editing. TS: Formal Analysis, Writing – review & editing. SF: Formal Analysis, Writing – review & editing. NM: Formal Analysis, Methodology, Writing – review & editing. MB: Formal Analysis, Methodology, Writing – review & editing. BS: Formal Analysis, Writing – review & editing. FL: Conceptualization, Supervision, Validation, Visualization, Writing – review & editing. EL: Funding acquisition, Project administration, Supervision, Writing – review & editing. CP: Conceptualization, Project administration, Supervision, Writing – review & editing. **IT:** Data curation, Funding acquisition, Project administration, Supervision, Validation, Writing – review & editing.

## Supplementary material

Supplementary Figure 1 Illustration depicting the comparative results between the ACO group and the asthma group (OVA group), as well as between the ACO group and the COPD group (PPE group).
