# Supplementary figures and images for: Modulating asthma–COPD overlap responses with IL-17 inhibition
Source: Front Immunol. 2023 Oct 27;14:1271342. doi: 10.3389/fimmu.2023.1271342 (PMC10641519; doi:10.3389/fimmu.2023.1271342)

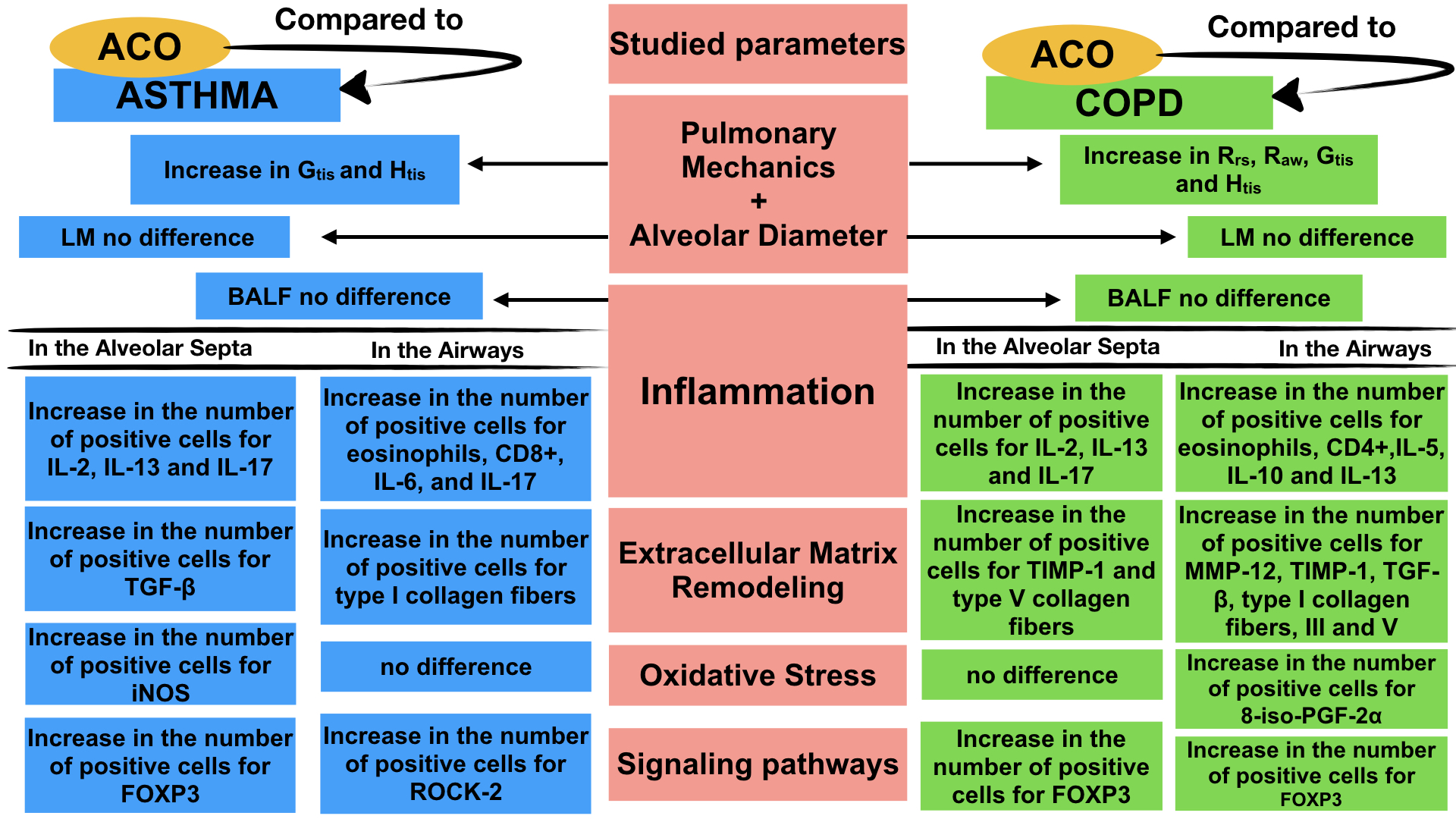

Supplement: Supplementary Figure 1 — Illustration depicting the comparative results between the ACO group and the asthma group (OVA group), as well as between the ACO group and the COPD group (PPE group). [file Image_1.jpeg]
